# Development and validation of an advanced fragment analysis‐based assay for the detection of 22 pathogens in the cerebrospinal fluid of patients with meningitis and encephalitis

**DOI:** 10.1002/jcla.22707

**Published:** 2019-01-21

**Authors:** Fang Long, Mimi Kong, Siying Wu, Weili Zhang, Quanfeng Liao, Zaisheng Peng, Li Nan, Ya Liu, Minjin Wang, Chao He, Yong Wu, Xiaojun Lu, Mei Kang

**Affiliations:** ^1^ Department of Laboratory Medicine, West China Hospital Sichuan University Chengdu China; ^2^ Ningbo HEALTH Gene Technologies Co., Ltd. Ningbo China; ^3^ Enshi Tujia and Miao Autonomous Prefecture Center for Disease Control and Prevention Enshi China

**Keywords:** advanced fragment analysis, encephalitis, meningitis, pathogen

## Abstract

**Background:**

Meningitis and encephalitis (ME) are central nervous system (CNS) infections mainly caused by bacteria, mycobacteria, fungi, viruses, and parasites that result in high morbidity and mortality. The early, accurate diagnosis of pathogens in the cerebrospinal fluid (CSF) and timely medication are associated with better prognosis. Conventional methods, such as culture, microscopic examination, serological detection, CSF routine analysis, and radiological findings, either are time‐consuming or lack sensitivity and specificity.

**Methods:**

To address these clinical needs, we developed an advanced fragment analysis (AFA)‐based assay for the multiplex detection of 22 common ME pathogens, including eight viruses, 11 bacteria, and three fungi. The detection sensitivity of each target was evaluated with a recombinant plasmid. The limits of detection of the 22 pathogens ranged from 15 to 120 copies/reaction. We performed a retrospective study to analyze the pathogens from the CSF specimens of 170 clinically diagnosed ME patients using an AFA‐based assay and compared the results with culture (bacteria and fungi), microscopic examination (fungi), polymerase chain reaction (PCR) (*Mycobacterium tuberculosis*), and Sanger sequencing (virus) results.

**Results:**

The sensitivity of the AFA assay was 100% for 10 analytes. For *Cryptococcus neoformans*, the sensitivity was 63.6%. The overall specificity was 98.2%. The turnaround time was reduced to 4‐6 hours from the 3‐7 days required using conventional methods.

**Conclusions:**

In conclusion, the AFA‐based assay provides a rapid, sensitive, and accurate method for pathogen detection from CSF samples.

## 1 INTRODUCTION

Meningitis and encephalitis (ME) are severe central nervous system (CNS) infections with high morbidity and mortality because of the difficulty in achieving a prompt diagnosis and receiving timely therapy.^1^ CNS infections are mainly caused by pathogens including bacteria, mycobacteria, fungi, viruses, and parasites.^2^ Cerebrospinal fluid (CSF) culture is necessary to diagnose ME.^3^ CSF culture is the gold standard for the diagnosis of bacterial infections, though it is time‐consuming. Microscopic examination, blood culture, skin biopsy, and serum inflammatory markers are additional diagnostic tools that might aid in etiological diagnoses. If the causative pathogen is not clear, the clinician will preliminary determine the type of pathogen (bacteria, virus, or fungus) according to the patient's clinical manifestations and the cellular and chemical parameters of the CSF. All of these laboratory tests require a certain CSF volume. However, the methods listed above are time‐consuming and generally have low sensitivity or specificity. In recent decades, the epidemiology and treatment strategies for meningitis have changed considerably, especially because of the introduction of conjugate vaccines such as the vaccines for *pneumococcal*, *meningococcal, *and *Haemophilus influenza* type b.^4^, ^5^, ^6^, ^7^, ^8^ Therefore, the early diagnosis of ME has become even more imperative. Doctors may sometimes perform comprehensive anti‐infection therapy, including antibiotics and antiviral and antifungal medications, immediately for cases that lack a definitive pathogen diagnosis if the patients are critically ill. However, most such treatments are ineffective, and certain drugs might be harmful to patients. Therefore, there is an urgent need for a rapid, sensitive, and accurate method that can detect a greater number of target pathogens from a small CSF volume.

According to population‐based studies in China, the incidence of acute bacterial meningitis ranges from 12.4 to 19.2 cases/100 000 for children aged <5 years.^9^, ^10^, ^11^ The primary pathogens of bacterial meningitis are *Neisseria meningitidis*, *H. influenza*, and *Streptococcus pneumonia.* 12, 13, 14 China has the second‐highest prevalence of tuberculosis (TB) infection worldwide. China and another 21 high‐burden countries account for 80% of the tuberculosis cases and approximately 22% of multidrug‐resistant tuberculosis cases worldwide.^15^, ^16^ Tuberculous meningitis (TBM) is the most severe form of extrapulmonary tuberculosis (EPTB) and causes exceptionally high mortality and morbidity.^17^, ^18^, ^19^ Viruses are the major cause of aseptic meningitis. Human enteroviruses (HEVs) are a common cause of acute meningitis with a summer‐fall season peak. Yihong Xie et al reported a 5‐year study on acute ME in Guangxi, China. Their study revealed that enterovirus (31.5%) is the most common pathogen, followed by Japanese encephalitis (28.3%), mumps (23.2%), measles (5.1%), herpes simplex virus, rubella, cytomegalovirus, varicella zoster virus and EB virus.^20^ Among the fungi, *Cryptococcus neoformans* is the most common cause of fungal meningitis.

Advanced fragment analysis (AFA) is a technique that provides an alternative high‐throughput, multiplexed, quantitative gene expression analysis method. AFA is based on the design of primers for different targets. The products obtained by amplification of each target point should have a size difference of at least 4 bp. The primers are synthesized and then fluorescently labeled at the end. Thus, fragments with different sizes are fluorescently labeled with different colors. Multiple polymerase chain reactions (PCRs) are performed to ensure that all nucleic acid fragments are fluorescently labeled. After pretreatment, the product is subjected to capillary electrophoresis; products with different fragment sizes are identified and distinguished by a fluorescence detector. Software is used to analyze the data and determine the size and genotype. Traditional detection methods, such as culture, require at least 2‐3 days. Currently, commonly used molecular detection methods, such as RT‐PCR, can only detect 1‐2 pathogens in one experiment, but AFA can detect up to 40 pathogens in 4‐6 hours. Thus, AFA has an absolute advantage in terms of the detection time and number of pathogens. AFA has been successfully applied in many fields, such as subtype classification of pediatric acute lymphoblastic leukemia, detection of multiple viruses for community‐acquired pneumonia, *Helicobacter pylori* identification, and virulence and resistance analyses.^21^, ^22^, ^23^

In the present study, the AFA technology was used to design primers for 22 target pathogens based on national epidemiological data. The most commonly reported pathogens causing ME, including eight viruses, 11 bacteria, and three fungi, were selected as detection targets. The targets were divided into panels A and B that comprise 13 and nine targets, respectively. Panel A includes *Escherichia coli*, *Mycoplasma pneumoniae*, *Mycobacterium tuberculosis*, *N. meningitidis*, *Streptococcus pneumonia*, *Staphylococcus*, cytomegalovirus (CMV), enterovirus (EV), Epstein‐Barr virus (EBV), herpes simplex virus type 1 (HSV‐1), herpes simplex virus type 2 (HSV‐2), varicella zoster virus (VZV), and* C. neoformans*. Panel B includes *Acinetobacter baumanii*, *Haemophilus influenzae*, *Listeria monocytogenes*, *Nocardia*, *Streptococcus agalactiae*, human herpes virus type 6 (HHV‐6), mumps virus (MuV), *Cryptococcus laurentii*, and *Cryptococcus albidus*.

In this retrospective study, residual CSF specimens were collected and tested at the West China Hospital of Sichuan University. The results of the AFA‐based assay were compared with those of conventional culture for bacteria and yeast, PCR for *M. tuberculosis*, and Sanger sequencing for viruses.

## 2 MATERIALS AND METHODS

### 2.1 Study design and case definition

This study was a retrospective study of 170 patients admitted to the hospital for the first time due to ME. Meningitis was defined as an infection localized to the subarachnoid space sparing the brain parenchyma and was characterized by a fever, headache, nausea, vomiting, meningeal irritation, and alterations in the CSF.^24^ Encephalitis was defined as the presence of an inflammatory process in the brain associated with clinical evidence of neurologic dysfunction.^25^ The ME patients were hospitalized at West China Hospital of Sichuan University from January 2016 to November 2016. The study protocol was approved by the Biomedical Ethics Committee of the West China Hospital of Sichuan University. The Ethics Approval Number is 203 (2015).

The criteria of inclusion and exclusion for the present study: (a) The patients were clinically diagnosed with ME. (b)The patient's CSF specimens were meeting the following clinical specimen criteria. (c) The patients were recurrent or review ME or diagnosed with carcinomatous meningitis, leukemia meningitis, anti‐NMDA (N‐methyl‐D‐aspartate) receptor encephalitis and have incomplete medical records were excluded.

### 2.2 Clinical specimens

Specimens meeting the following inclusion criteria were selected: A CSF specimen was collected by lumbar puncture (LP) with adequate residual volume of uncentrifuged CSF (≥200 µL) left over from standard care testing for bacterial and yeast culture, and the specimen was enrolled within 7 days of collection for testing (<5 days frozen for nucleic acid extraction and 2 days for final testing). Each residual specimen collected for the study was assigned a unique number corresponding to our laboratory tests for the AFA assay. Thus, the authors had access to information that could not identify individual participants during or after data collection, including comparator PCR and patient demographic and clinical data, such as patient general information, CSF chemistry results (white blood cell [WBC] count and differential [if performed], protein, glucose, and chlorinate), lumbar puncture (LP), opening pressure and closing pressure, radiological findings (computed tomography [CT] and magnetic resonance imaging [MRI]), any additional CSF tests, the final CSF bacterial culture, yeast culture, and ink staining. Additionally, the hospitalization time, clinical features, clinical signs, immune status of the subject, and final clinician diagnosis and prognosis were recorded. The clinician diagnosed ME based on the above information.^26^

### 2.3 DNA/RNA extraction

DNA and RNA were extracted and purified using the ZD‐XJ‐Mini‐50 nucleic acid extraction kit (ZD Biotech, Ningbo, China) according to the manufacturer's instructions. The CSF samples were stored in a −80°C freezer until use (<5 days). Total nucleic acid extraction using 200 µL of the residual CSF was carried out immediately after thawing. The final nucleic acid sample was resuspended in 60 µL of DNase/RNase‐free water. The extracts were used immediately for PCR amplification or were stored at −20°C for weekly analysis.

### 2.4 Primer design

Target‐specific primers were designed based on the alignment of hundreds of target sequences from the National Center of Biotechnology Information (NCBI). Conserved regions were selected for primer design. Primers were evaluated using Primer Premier 5.0 (Premier Biosoft International, Palo Alto, CA, USA) and DNASTAR Lasergene software (DNASTAR Inc, Madison, WI, USA). Homologous regions prone to mispriming were excluded. The gene‐specific primers were designed to yield PCR fragments at least four base pairs (bp) apart, ranging from 109 to 313 bp. The reverse primers were labeled with 6‐carboxyfluorescein (FAM) reporter dye at the 5′‐end. Information for all of the primers is listed in Tables S1 and S2. All primers were synthesized by Sangon Biotech (Shanghai, China).

### 2.5 AFA‐based multiplex assay

An RT‐PCR mixture containing 4.5 µL of premixed solution, 0.5 µL of an RT‐PCR enzyme and UDG enzyme mixture, and 5 µL of sample or positive control or negative control was added to a final volume of 10 µL/reaction. PCR amplification was performed using the ABI Verity 96 Thermal Cycler. The cycling conditions are listed in Table S3.

Polymerase chain reaction products were prepared for capillary electrophoresis (CE) and fragment analysis using the 3500 Genetic Analyzer (ABI, USA) following the manufacturer's protocols. Next, 1.0 µL of PCR product was added to 10 µL of Hi‐Di formamide solution along with 0.25 µL of GeneScan™‐500 LIZ™ Size Standard (ABI, Foster City, CA, USA). The mixture was added to a 96‐well plate, which was loaded onto a 3500 Genetic Analyzer for CE and fragment separation. The fragment size was used for target identification. For all targets, the assay was considered positive when the signal strength of the fluorescent dye was above 500 relative fluorescence units (RFU), undetermined for a signal strength between 300 and 500 RFU, and negative for a signal strength <300 RFU. If the signal strength was in the undetermined region, a repeat test was performed; if the test result was still in the undetermined zone, it was deemed positive. The corresponding amplicon sizes of the pathogens are listed in Tables S1 and S2.

In addition, each panel incorporated three reference genes, including B2M and RNaseP to monitor the quality of the extracted mRNA and DNA, respectively. For the B2M gene, the assay was designed to amplify mRNA around the second and third intron‐exon junction to ensure that the mRNA was amplified. Additionally, an internal control (IC) was included as a quality control for the RT‐PCR reaction. The One‐Step RT‐PCR Kit used for multiplex pathogen detection was obtained from Health Gene Technologies Co., Ltd. (Ningbo, China).

### 2.6 AFA ME panel testing

The AFA ME panel test consisted of nucleic acid extraction (50 minutes), reverse transcription and nucleic acid amplification (140 minutes), and fragment analysis (50 minutes), as shown below. All of the above steps plus the manual operation time (approximately 30 minutes) required 4‐6 hours.

### 2.7 Sensitivity of the AFA‐based assay

For sensitivity studies, a recombinant plasmid was used. The RT‐PCR products were extracted from a 1% agarose gel and then purified using a Gel Extraction Kit D2500 (OMEGA Bio‐Tek, Norcross, GA, USA). The purified PCR product was ligated to the pMD® 18‐T Simple Vector, which was used to transform *E. coli* (DH5a). White colonies were picked and inoculated in LB medium containing ampicillin and then were cultured overnight at 37°C. PCR was used to identify the cloned bacteria. The plasmid was extracted from *E. coli* using a Plasmid Mini Kit I D6943 (OMEGA Bio‐Tek). The extracted plasmid was digested with EcoRI and HindIII. The positive recombinant plasmid was sequenced (Sangon Biotech) and identified using the BLAST tool of NCBI. After successful construction of the recombinant plasmid, each target pathogen recombinant was measured via twofold serial dilutions in PBS. The copy numbers were determined using the following formula: number of copies = (DNA amount * 6.022 × 10^23^)/(DNA length * 1 × 10^9^ * 660); number of copies = (ng * number/mole)/(bp * ng/g * g/mole of bp). The diluted plasmid was used for the determination of the limit of detection.

For specificity, we used four clinically isolated strains (*Enterobacter cloacae*, *Klebsiella pneumoniae*, *Pseudomonas aeruginosa*, and *Candida albicans*) that often appear in the CSF of ME patients and were not included in the AFA panel. We extracted nucleic acids and then mixed samples of those four pathogens with CMV DNA in PBS, followed by detection using the AFA assay.

### 2.8 Comparator testing

Bacterial and fungi cultures were performed on each specimen enrolled. Each sample was inoculated onto a blood agar plate, a chocolate agar plate, and a Sabouraud agar plate as well as in brain heart infusion broth (Autobio, Zhengzhou, China). These plates and broth were then incubated overnight at 37°C (except for the Sabouraud agar plate, which was incubated at 25°C) and in 5% CO_2_ for 30 days. The suspected* M. tuberculosis* samples were cultured using Middlebrook 7H9 broth and Lowenstein Jensen culture medium at 37°C for 42 days. Tests were performed using the laboratory's standard operation procedures. Positive growth was recorded and identified using the API 20 C system (BioMerieux, Marcy L'Etoile, France) and matrix‐assisted laser desorption ionization‐time of flight mass spectrometry (MALDI‐TOF MS) analysis (Bruker, Bremen, Germany). The accuracy of all tests was confirmed by proficiency testing by the College of American Pathologists.

Direct microscopic examination was performed using cytocentrifuged CSF, including CSF Jincheng ink staining (Yin Teli Stationery Co, Sichuan, China), Gram staining (BioMerieux) and acid‐fast staining (Howsome, Shanghai, China).

For *M. tuberculosis *detection*,* DNA was extracted from patients with clinically diagnosed tuberculous meningitis (TBM) using the MagNA Pure LC 2.0 automated system with the total nucleic acid isolation high‐performance kit (Roche Diagnostics, Indianapolis, IN, USA). Next, the samples were subjected to RT‐PCR for MTB DNA using a commercial kit (Qiagen, Hilden, Germany) that was previously included in the diagnostic criteria for TBM.^19^, ^27^

For viral detection, samples for sequencing were extracted using AFA nucleic acid. All of the clinically confirmed positive samples were sequenced by Sangon Biotech, and then, the data were compared with the information of NCBI to assess whether the requirements were met.

### 2.9 Results and discrepant analysis

The AFA assay result was considered true positive (TP) or true negative (TN) only when it agreed with the result from the comparator method. Additionally, the discrepancy investigation for our study relied heavily on additional clinical information about the subjects whose specimens were tested in this evaluation.

### 2.10 Calculations and statistical analysis

Sensitivity and PPA were calculated as 100 × [TP/(TP + FN)], and specificity and NPA were calculated as 100 × [TN/(TN + FP)]. As described previously, PPA and NPA were calculated in the same manner as those for the sensitivity and specificity, respectively. The terms “PPA” and “NPA” are used instead of “sensitivity” and “specificity” to indicate that a non–gold standard assay (eg, PCR) was used for the original comparator analysis. This analysis referenced the study by Leber et  al^28^ concerning the evaluation of the BioFire FilmArray Meningitis/Encephalitis Panel.

## 3 RESULTS

### 3.1 Group assembly

After screening 234 patients clinically diagnosed with ME, we excluded 64 patients for the following reasons: recurrent (n = 12) or review ME (n = 10), diagnosed with carcinomatous meningitis or leukemia meningitis (n = 7), diagnosed with anti‐NMDA‐receptor encephalitis (n = 15), and incomplete medical records (n = 20). Therefore, a total of 170 patients were enrolled and divided into five groups based on the clinicians’ diagnoses as follows: bacterial group (except tuberculous), viral group, fungal group, tuberculous group, and undefined group. The definition of each subgroup was referred to in the previous reference, and their precise definitions are provided in reference Table S6.^25^, ^26^, ^29^, ^30^

### 3.2 Specificity, accuracy, and study of the AFA‐based assay

The primer specificity of the multiplex AFA‐based assay was verified by Sanger sequencing. Twenty‐two clinically confirmed pathogens causing ME were used to evaluate the accuracy of the assay. The multiplex study used the positive clinical samples, and positive controls consisted of recombinant plasmids. The multiplex study of clinical samples showed specific peaks for all three references (Hu_RNA, Hu_DNA, and IC), the positive controls, and pathogens in the panel. The AFA‐based multiplex assay showed 100% agreement with the Sanger sequencing. The four clinically isolated strains (*E. cloacae*, *K. pneumoniae*, *P. aeruginosa*, and *C. albicans*) did not produce any signals that would indicate nonspecific amplification when they were mixed with HCMV (figures shown in Figure S1A‐E).

The recombinant plasmids were used to determine the limit of detection. The cutoff value was 500 RFU for positivity. The limit of detection for each target in the current assay is listed in S4.

### 3.3 Clinical specimen demographics

We acquired a total of 170 retrospective CSF specimens that were clinically diagnosed with ME. The age distribution included 159 (93.5%) adults aged 16 years and 11 (6.5%) pediatric of patients aged <16 years.

The demographic data, clinical features, laboratory test results, and prognoses of the patients are shown in Tables S1 and S2. Regarding the data presented in the table, the number of males (101 [59.4%]) included in this study was slightly higher than that of females (69 [40.6%]). Most of the 170 patients with ME were of the Han ethnicity (151 [88.8%]), followed by the Tibetan ethnicity (16 [9.4%]) and others (3 [1.8%]). The Tibetan group was comprised mainly of TBM patients (12/14 [85.7%]). Twenty‐seven (15.9%) patients had basic illnesses of diabetes or hypertension, and 14 patients (9.3%) had immunocompromised conditions, including six patients with HIV or AIDS. Eighty (47.1%) patients had lung infection, 62 (36.5%) had hypokalemia, and 27 (15.9%) had hypoproteinemia. The clinical features and signs used to diagnose ME were fever (102 [60%]), headache (106 [62.4%]), vomiting (26 [15.3%]), nuchal rigidity or stiff neck (68 [40%]), meningeal irritation (26 [15.3%]), mental disorders (16 [9.4%]), seizures (46 [27.1%]), confusion (41 [24.1%]), and sinusitis or otitis (73 [43.0%]). The baseline characteristics of the 170 ME patients are shown in Table S5.

### 3.4 Laboratory examination of the CSF, serum, and radiological findings

Regarding the CSF analyses, the median CSF opening pressure was 180 mmH_2_O, and 32 (18.8%) showed an increased CSF opening pressure (>250 mmH_2_O). The Fungal ME CSF opening pressure (240 [190‐320] mmH_2_O) was slightly higher than TBM (210 [90‐350] mmH_2_O) and BM (200 [30‐280] mmH_2_O). Viral ME (120 [61‐350] mmH_2_O) were less common than the other types in 170 patients, of whom 158 had CSF routine analysis, 124 (78.5%) had CSF clear appearance, and the median CSF leukocyte count was 70 * 10^6^/L, with that in the BM group (670 × 10^6^/L) considerably higher than in the other groups. The median CSF glucose concentration was 2.74 (0.02‐7.96) mmol/L. In the BM group, the percentage of patients who had a decreased glucose concentration (<2.5 mmol/L), the level of patients' CSF protein >0.45 g/L, the CSF IgG synthesis rate, and the C‐reactive protein were much higher than in the other groups. Regarding the radiological findings, 75 (47.47%) and 109 (68.13%) patients had undergone head CT and MRI, respectively. Additionally, 45 (41.28) patients who had undergone MRI showed meningeal enhancement. The MRI (53.2%) abnormal rate was slightly higher than that of CT (30.7%), especially for the bacterial, tuberculous, and fungi groups. The laboratory examinations of CSF and sera and the radiological findings are shown in Table S6.

### 3.5 Summary of the AFA panel findings for the clinical samples

The AFA ME panel detected at least one potential pathogen in 50 of the 170 specimens that were tested, shown as a positivity rate of 29.4% in Table 1. The highest detection rates were in the pediatric groups.

**Table 1:** Positivity rate for the AFA ME panel for all ME patients and by age group

Parameter	Positivity	% of total
	No. of samples	
Negative samples	120	70.5
Positive samples	50	29.5
Single detection	48	28.2
Codetections	2	0.3
Age group in years (n)		
5‐10 (2)	1	50.0
11‐15 (9)	4	44.4
16‐36 (60)	18	30.0
37‐60 (68)	20	29.4
>61 (31)	7	22.6

### 3.6 Summary of AFA and the comparator test findings

For the 170 clinical samples, both methods detected 36 target pathogens at the same time. The results were consistent. A total of 17 pathogens were positive in the AFA and negative in the comparator method. Four fungal pathogens were detected with the comparator method but were negative with the AFA, as shown in Table 2.

**Table 2:** AFA and comparator testing results

AFA	Comparator testing		Total
	+	−	
+	37 (six bacterial, 12 TB, 12 viral, seven fungal)	16 (10 bacterial, two TB, four viral)	53
−	Four fungal	116	120
Total	41	132	173^a^

a Since two CSF samples had detection of two pathogens and three pathogens at the same time, the total number at this site is 170 − 2 + 5 = 173.

### 3.7 Summary of AFA ME panel findings

*Mycobacterium tuberculosis*, *C. neoformans*, *EBV*, *A. baumanii, *and *E. coli *were found in 14 (26.4%), seven (13.2%), six (11.3%), five (9.4%), and four (7.5%) specimens, respectively. All other assay targets were detected in three (5.7%) or fewer of the specimens. *M. pneumoniae*, *Nocardia*, *H. influenzae*, *L. monocytogenes*, *S. agalactiae*, HSV‐2, HSV‐6, *Cryptococcus gattii, *and *C. albidus *were the nine targets with no AFA detection in this study, as shown in Table 3.

**Table 3:** Total number of AFA assay detections by total positive detections

Target	No. detected	% of positive samples
Bacteria		
*Acinetobacter* * baumanii*	5	9.4
*Escherichia* * coli*	4	7.5
*Haemophilus* *influenzae*	0	0
*Listeria* *monocytogenes*	0	0
*Mycoplasma* * pneumoniae*	0	0
*Mycobacterium* * tuberculosis*	14	26.4
*Nocardia*	0	0
*Neisseria* * meningitidis*	2	3.8
*Staphylococcus*	3	5.7
*Streptococcus* *agalactiae*	0	0
*Streptococcus* *pneumonia*	2	3.8
Total	30	56.6
Viruses		
CMV	3	5.7
EBV	6	11.3
EV	3	5.7
HSV‐1	2	3.8
HSV‐2	0	0
HHV‐6	0	0
MuV	1	1.9
VZV	1	1.9
Total	16	30.2
Yeasts		
*Cryptococcus* * gattii*	0	0
*Cryptococcus* * albidus*	0	0
*Cryptococcus* * neoformans*	7	13.2
Total	7	13.2

CMV, cytomegalovirus; EBV, Epstein‐Barr virus; EV, enterovirus; HSV, herpes simplex virus; MuV, mumps virus; VZV, varicella zoster virus.

Codetections were observed in two specimens representing 1.2% of the specimens and 4% of the positive specimens (2/50), as shown in Table 1. The codetections were as follows: CMV and *A. baumanii* and *M. tuberculosis*, *N. meningitidis*, and EBV.

The summary of performance characteristics for each AFA assay target is presented in Table 4. The sensitivity/PPA and specificity/NPA were calculated with respect to the comparator test. The AFA assay demonstrated a sensitivity/PPA of 100% for 10 of the 22 analytes: *E. coli,* *A. baumanii*, *Staphylococcus*, *M. tuberculosis*, CMV, MuV, EBV, VZV, HSV‐1, and EV. One analyte had lower sensitivity (63.6% for *C. neoformans), *and *S.* *pneumonia* and *N. meningitidis* had 0% sensitivity without culture. Nine analytes were not detected by the AFA assay; therefore, no sensitivity could be calculated (*M. pneumoniae*, *Nocardia*, *H. influenzae*, *L. monocytogenes*, *S. agalactiae*, HSV‐2, HSV‐6, *C. gattii*, and *C. albidus*). The specificity/NPA of the AFA assay was 98.2% or greater for all analytes.

**Table 4:** Performance summary and characteristics of the AFA assay versus those of the comparator test

Target	Sensitivity/PPA		Specificity/NPA	
	TP/(TP + FN)	%	TN/(TN + FP)	%
Bacteria				
*Acinetobacter* * baumanii*	4/4	100	165/166	99.4
*Escherichia* * coli*	1/1	100	166/169	98.2
*Haemophilus* *influenzae*	0/0		170/170	100
*Listeria* * monocytogenes*	0/0		170/170	100
*Mycoplasma* * pneumoniae*	0/0		170/170	100
*Mycobacterium* * tuberculosis*	12/12	100	156/158	98.7
*Nocardia*	0/0		170/170	100
*Neisseria* * meningitidis*	0/0	0	168/170	98.8
*Staphylococcus*	1/1	100	167/169	98.8
*Streptococcus* *agalactiae*	0/0		170/170	100
*Streptococcus* *pneumonia*	0/0	0	168/170	98.8
Viruses				
CMV	3/3	100	167/170	98.2
EV	2/2	100	167/168	99.4
EBV	3/3	100	164/167	98.2
HSV‐1	2/2	100	168/168	100
HSV‐2	0/0		170/170	100
HSV‐6	0/0		170/170	100
MuV	1/1	100	169/169	100
VZV	1/1	100	169/169	100
Yeasts				
*Cryptococcus* * gattii*	0/0		170/170	100
*Cryptococcus* * albidus*	0/0		170/170	100
*Cryptococcus* * neoformans*	7/11	63.6	159/159	100

CMV, cytomegalovirus; EBV, Epstein‐Barr virus; EV, enterovirus; HSV, herpes simplex virus; MuV, mumps virus; VZV, varicella zoster virus.

## 4 DISCUSSION

With the worldwide change in the epidemiology of ME pathogens and universal use of antibiotics, as well as the application of multivalent combination vaccines, the clinical presentation of many ME cases is nonspecific, making a definitive etiologic diagnosis challenging.^31^, ^32^ The diagnosis of ME infections requires consideration of the most likely causative agents based on exposure, geography, and season as well as an understanding of the optimal diagnostic test and highest‐yield clinical specimen or testing.^33^, ^34^ It is particularly important to develop rapid detection reagents that could detect many pathogens in one PCR for ME patients who use only a small CSF sample value.

It is generally accepted that early, accurate medication is correlated with a better prognosis of patients. A previous study has shown that delayed treatment significantly increases the risk of a fatal outcome, with a relative increase in mortality of 12.6% per hour of delay.^35^ The delay is primarily associated with difficulties in recognizing ME due to the absence of typical symptoms in many cases.^31^, ^32^

Regarding conventional methods, CSF culture is still the gold standard for the diagnosis of CNS infections, especially for bacteria and fungi. However, the yield of CSF cultures in suspected ME cases is low, especially if the patients have received antibiotics. Furthermore, most CSF cultures require up to 72 hours for final identification.^36^

Regarding microscopic examination, the reported sensitivities vary for different microorganisms.^37^, ^38^, ^39^ A reported sensitivity of Gram staining for the diagnosis of bacterial meningitis was 60%‐80% in patients who had not received antibacterial treatment and 40%‐60% among those on antibacterial treatment.^40^ Typically, *M. tuberculosis* and *C. neoformans *can be diagnosed by microscopy, which has satisfactory specificity but relatively poor sensitivity. Among the antigens used for meningitis assays, cryptococcal antigen is the most widely used. Recent reports have revealed the potential application of the detection of *M. tuberculosis*‐specific antigens in CSF for the rapid diagnosis of TBM.^41^, ^42^ For serology assay of viruses, the most definitive diagnosis of virus infections is established by detecting CSF IgM antibodies or demonstrating at least a 4‐fold increase in neutralizing antibody titers between the acute and convalescent phases.^43^, ^44^ CSF IgM is the most widely used test for HSV, CMV, and VZV.^43^ However, this antibody has high cross‐reactivity with other clinically relevant viruses and related vaccines.^44^ Regarding the detection of viruses, molecular testing has improved sensitivity and is faster than culture; thus, this technique has become the standard of care for many viral CNS infections, including HSV, EV, and human parechovirus infections.^45^ In addition, blood culture and histopathologic examination are complementary methods for the diagnosis of ME.

ME patient demographic data. In our study, 170 patients were clinically diagnosed with ME. These patients were mainly of the Han ethnicity (88.8%), followed by the Tibetan ethnicity (9.4%). We found an interesting phenomenon in that the Tibetans were mainly diagnosed with TBM (12/16 [75%]). Although we cannot explain why the patients of Tibetan ethnicity were more susceptible to TBM, we speculate that this susceptibility may be related to a genetic factor, but this issue requires further study. Regarding cryptococcal meningitis (CM), in other countries, such as the United States and Brazil, previous studies showed that 79.4% and 95% of patients, respectively, were HIV infected. However, in China, CM occurs most commonly in HIV‐uninfected patients, a finding that is consistent with our data in this study (3/14 [21.4%]) and our group's previous study.^46^, ^47^, ^48^ The most common clinical features and signs in those patients were headache, fever, sinusitis or otitis, seizures, and confusion. Only fever and headache occurred in more than half of the patients, and some patients were finally diagnosed with ME with no obvious clinical manifestations. In addition to CSF culture, microscopic examination, and other CSF‐related laboratory tests, radiological findings were a good supplement to the diagnosis of ME.

### 4.1 AFA assay of bacterial targets

Sixteen targets were detected by AFA other than TB. Six specimens (37.5%) were also positive with culture. No *M. pneumoniae*, *Nocardia*, *H. influenzae*, *L. monocytogenes*, and *S. agalactiae *were detected using the tests; thus, sensitivity calculations were not possible*.* Ten specimens for bacterial targets were AFA assay‐positive and culture‐negative at the same time. The clinical and laboratory results of six patients with inconsistent results strongly supported the AFA findings, including those of two *S. pneumonia* specimens, two *E. coli* specimens, one *Staphylococcus* specimen, and one *N. meningitidis* specimen. For one case of *E. coli*, the CSF smear was positive for a Gram‐negative bacillus.

There was no evidence to support the AFA assay results for the remaining four bacterial results (one case each of *E. coli*, *S* *aureus*,* A. baumanii*, and *N. meningitidis*). The final diagnoses and CSF parameters of all four of these patients disagreed with the AFA assay results. The patient who was positive for *E. coli* detection was a 49‐year‐old woman who had CSF pleocytosis (90 × 10^6^/L) with mononuclear predominance (54%) and a final diagnosis of TBM (patient 43 in Table S7). The patient who was positive for *Staphylococcus* detection was a 23‐year‐old man who had normal CSF parameters and a final diagnosis of viral ME (patient 63 in Table S7). The patient with *A. baumanii* detection was a 29‐year‐old man with a final diagnosis of TBM (patient 108 in Table S7). The results for the patient with *N. meningitidis*,* M. tuberculosis*, and EBV detection were highly suspected of contamination with *N. meningitidis*, because the final diagnosis was TBM. However, the patient's serum EBV PCR test was positive. Additional data for all inconsistent results with associated laboratory data and a final clinical diagnosis are presented in Table S7 in the Supporting Information.

Regarding TBM, we used culture and PCR as the comparator assay. The AFA assay detected 14 *M. tuberculosis* cases. Twelve cases were also positive by culture or PCR, and two specimens were inconsistent; other data supported the AFA assay results. Among the 12 consistent *M. tuberculosis* specimens, six (50%) showed other types of TB infection (ie, tuberculosis, genital tuberculosis, and abdominal pelvic tuberculosis). Eleven patients underwent the IGRA test, with 10 (90.9%) testing positive. The two patients with inconsistent TB detection were a 68‐year‐old male and a 15‐year‐old male (patients 5 and 159, respectively, in Table S7). Both showed a positive IGRA test, and one of them had genital tuberculosis. Two patients had abnormal MRI results—ring enhancement and nodular enhancement, respectively—highly suggestive of patients with TBM.

Although our AFA panel included the most common causative agents for bacterial targets in China, it could not cover all possible agents that cause bacterial CNS infections. Therefore, traditional methods of detection, such as culture, microscopic examination, blood culture, serological examination, and pathological examination, cannot be completely replaced by AFA testing. In our study, three bacterial strains reported from culture were not targeted by the AFA panel: one *Enterobacter sakazakii*, one *Pneumonia klebsiella*, and one *P. aeruginosa*. This result shows that although molecular methods require less time and are more sensitive, traditional methods are still indispensable.

### 4.2 AFA panel viral targets

Viral detection using the AFA assay was lower than the detection of bacterial targets. The sequencing comparator method confirmed the AFA assay results in 12 (75%) of these cases. The four inconsistent viral results were associated mainly with EBV infection (3/4 75%). The calculated PPA was very good for all targets (100%), except for HSV‐2 and HSV‐6, which were not detected; thus, their PPA could not be calculated. The inconsistent result for the EV‐positive case was obtained for a 61‐year‐old male with a final diagnosis of TBM. His clinical data, presentation, symptoms, and laboratory findings all confirmed the clinician's diagnosis (patient 12 in Table S7). For the three inconsistent EBV‐positive patients, two of three of their clinical and laboratory data did not support the AFA results. Thus, we speculated that the clinical significance of detecting EBV in the CSF was not clear and that the test most likely detected latent virus from cells that were present.

The above data show that the AFA assay has high sensitivity and specificity for the detection of viral pathogens and has a high application value, especially for laboratories without conventional PCR detection.

### 4.3 AFA panel fungal targets

Seven *C. neoformans*‐positive specimens were detected via the AFA assay, all of which were confirmed by culture and CSF Jincheng ink staining. The calculated NPA was 100%, and the PPA was 63.6%, which was lower than that of the FilmArray panel (100%).

For four cases of cryptococcals, CSF culture or CSF Jincheng ink staining was positive and the AFA was negative; all four patients had a final diagnosis of cryptococcal meningitis. Four patients with CSF Jincheng ink staining were positive several times, and for two of them, cultures of the CSF specimens collected at other periods were positive. A previous study reported similar results.^49^ This finding may be due to the fungal cell walls being more difficult to break, leading to low nucleic acid extraction efficiency. Additional data for all of the results with associated laboratory data and a final clinical diagnosis are presented in Table S7 in the Supporting Information.

After the comparative analysis, our study still had unresolved inconsistent results (n = 7), including four bacterial results and three viral results. We suspected that the main reasons for these unresolved results were as follows: (a) contamination from the sample collection process; (b) the risk presented by handling PCR products during the fragment analysis process; and (c) perhaps, the patient's clinical manifestation and laboratory tests were atypical and obvious (ie, in fact the patient had the pathogen infection detected by the AFA). The operator should wear a mask or respirator, and the test should be conducted in a biological safety cabinet to reduce the risk of contamination with respiratory flora from the operator. The final positive results require comprehensive clinical presentation, epidemiology, CSF routine analysis, radiological findings, any additional CSF tests, the final CSF culture, Gram staining, serology tests, blood culture, and other tests.

In conclusion, the AFA‐based assay is a rapid, sensitive, and specific method for detecting pathogens in CSF.

The method can also be employed as a supplement to the traditional methods for diagnosing ME. Accurate identification of causative pathogens causing ME will improve patient management and epidemiological investigations.

## Data Availability

All relevant data are included within the article and its Supporting Information files.
