# Supplementary material for: Development and validation of an advanced fragment analysis‐based assay for the detection of 22 pathogens in the cerebrospinal fluid of patients with meningitis and encephalitis
Source: J Clin Lab Anal. 2019 Jan 21;33(3):e22707. doi: 10.1002/jcla.22707 (PMC6818557; doi:10.1002/jcla.22707)
Supplement: Supplementary file 1 [file JCLA-33-e22707-s001.docx]

Figure S1A. AFA assay of CMV. CMV peaks and those of three internal references (Hu_RNA, Hu_DNA and IC) are shown on the electropherogram.


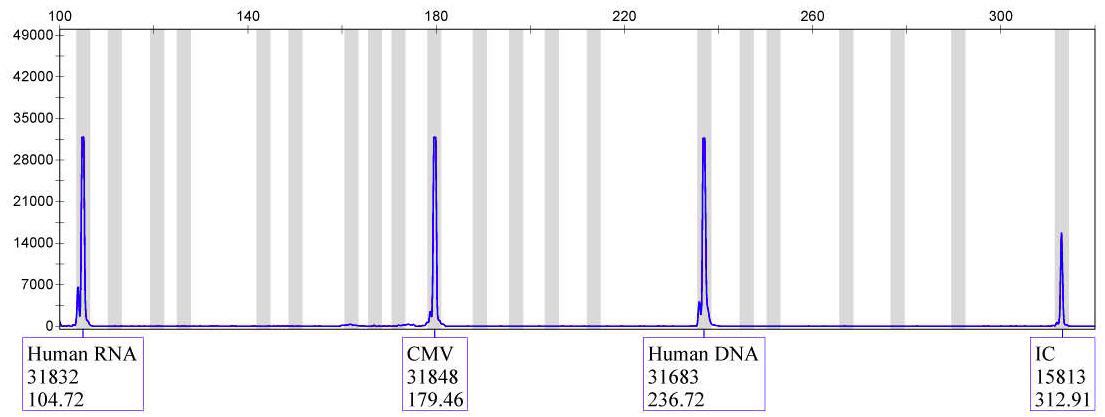


Figure S1B AFA assay of CMV mixed with *Enterobacter cloacae.* CMV peaks and those of three internal references (Hu_RNA, Hu_DNA and IC) are shown on the electropherogram.


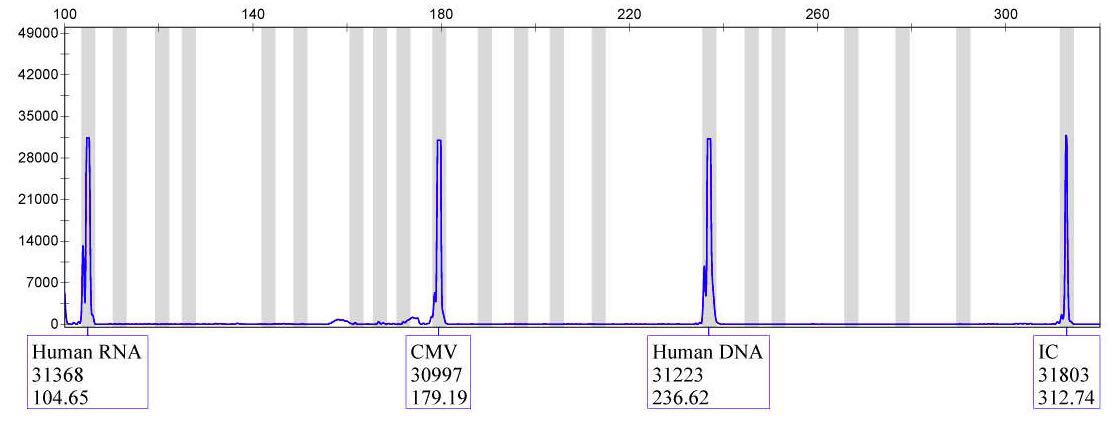


Figure S1C AFA assay of CMV mixed with *Klebsiella pneumoniae.* CMV peaks and those of three internal references (Hu_RNA, Hu_DNA and IC) are shown on the electropherogram.


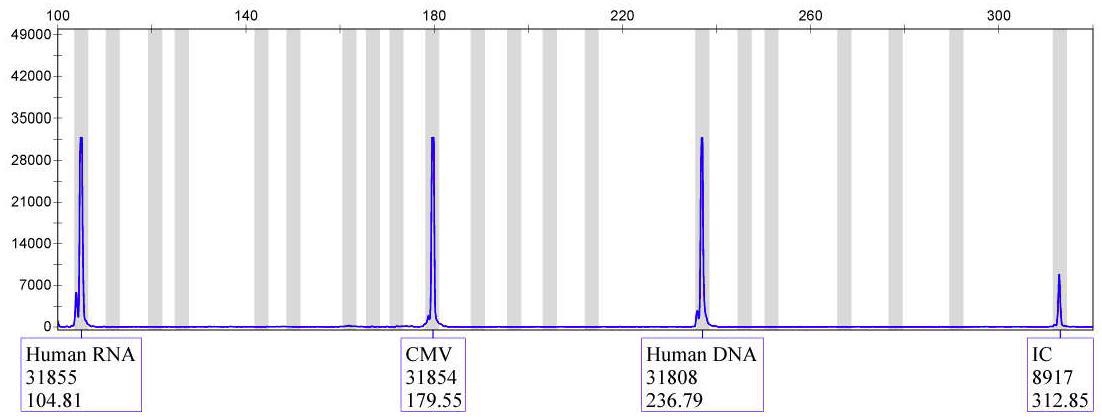


Figure S1D. AFA assay ofCMV mixed with *Pseudomonas aeruginosa.* CMV peaks and those of three internal references (Hu_RNA, Hu_DNA and IC) are shown on the electropherogram.


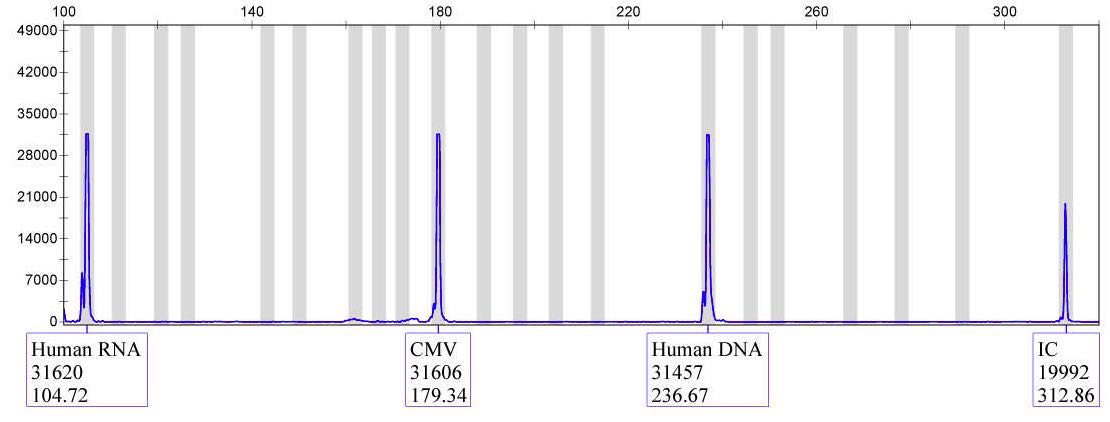


Figure S1E. AFA assay of CMV mixed with *Candida albicans.* CMV peaks and those of three internal references (Hu_RNA, Hu_DNA and IC) are shown on the electropherogram.


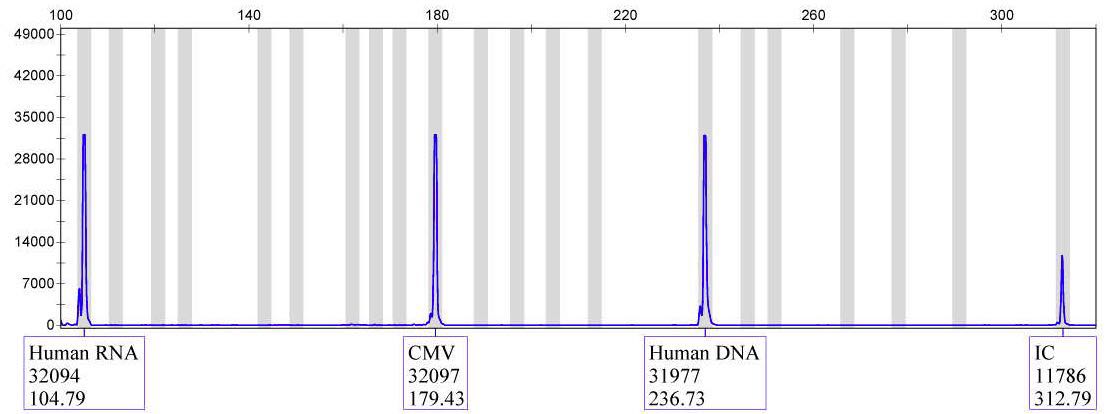


Supplementary Tables

Table S1. Primer Sequences and Product Sizes in Panel A

| Target | Amplicon Size (bp) | Sequence (5’-3’) | Accession number | Fluorescent |
| --- | --- | --- | --- | --- |
| Hu_RNA | 106 | GATGAGTATGCCTGCCGTG |  | N/A |
|  |  | ATGCGGCATCTTCAAACCT |  | FAM |
| EV | 109 | GCAATTCTACCATCACAACTCAGGA | AB192877.1 | N/A |
|  |  | CTAGTAGGCTKGTCCACTGCAG |  | FAM |
| *C. neoformans* | 115 | GTGGCGTTCACCAABGCAG | XM_012193014.1 | N/A |
|  |  | TACTTGTGTCTCCAGTTCTC |  | FAM |
| *N. meningitidis* | 120 | GCATCAGCCATATTCACACGATA | CP020423.1 | N/A |
|  |  | GTGTTCCGCTATACGCCATT |  | FAM |
| *S. pneumoniae* | 126 | GCGAACTCTTACGCAATCTAGCA | AP018044.1 | N/A |
|  |  | CGTGGTCTGAGTGGTTGTT |  | FAM |
| *Staphylococcus* | 140.3 | TATTAGCACTTGTMAGCACACCTTC | CP015817.1 | N/A |
|  |  | TGAACCTGGTGAAGTTGTRATCTG |  | FAM |
| EBV | 150 | GTGGACATTCTTCTGAAGGAACG | LC150743.1 | N/A |
|  |  | CGTCAGCAGACTAAGACTACTC |  | FAM |
| VZV | 160 | GATCTTGCTGATGCGGTTG | KY037798.1 | N/A |
|  |  | CCAATAGCAGAAGTGGAGGTT |  | FAM |
| HCMV | 179 | GCCTCTAYCCTTCCATCATC | KT726954.2 | N/A |
|  |  | CTTGTTGAGCAGTTCCGAAAG |  | FAM |
| TB | 192 | GTCAGGCAATGGCTTCGA | AP018036.1 | N/A |
|  |  | CGGAAGAGCTTGTCGTAGTC |  | FAM |
| HSV-1/2 | 196/205 | GTCGCCCTCCTCACAAACT | KX424525.1/ KX574908.1 | N/A |
|  |  | CACTTCCGTCCCCAATAAAC |  | FAM |
| *M. pneumoniae* | 213 | GCCAACAGTGAAACGAGGT | CP017341.1 | N/A |
|  |  | TTGCATCCTTCACCTTCA |  | FAM |
| *E. coli* | 230 | GCGTGGTTATCAGTTGGTGA | CP020835.1 | N/A |
|  |  | CAATGGCTTTRTCRGTCATCT |  | FAM |
| Hu_DNA | 237 | GGTGTTTGCAGATTTGGACCT |  | N/A |
|  |  | CGCTAGGAATCAGACCAACACCTC |  | FAM |
| IC | 313 | GTGGCCGCTTTTCTGGATTCAT |  | N/A |
|  |  | TGAAGGCACAGTCGAGGCTG |  | FAM |

EV: *enteroviruses*, HSV-1: *herpes simplex virus type 1*, HSV-2: *herpes simplex virus type 2*, VZV: *varicella zoster virus*, CMV: *cytomegalovirus*, EBV: *Epstein-Barr virus*, MP: *Mycoplasma pneumoniae*, *N. meningitidis: Neisseria meningitidis*, *S. pneumonia:* *Streptococcus pneumonia*, *M. tuberculosis: Mycobacterium tuberculosis*, *C. neoformans: Cryptococcus neoformans* and *E. coli: Escherichia coli*. Hu-RNA: Human RNA (B2M), Hu_DNA: Human DNA (RNaseP), IC: internal control.

Table S2. Primer Sequences and Product Sizes in Panel B

| Target | Amplicon Size (bp) | Sequence (5’-3’) | Accession number | Fluorescent |
| --- | --- | --- | --- | --- |
| Hu_RNA | 106 | GATGAGTATGCCTGCCGTG |  | N/A |
|  |  | ATGCGGCATCTTCAAACCT |  | FAM |
| *L. monocytogenes* | 144 | CACGAGAGCACCTGGATATG | CP020774.1 | N/A |
|  |  | GCAAATGTGCCGCCAAGAAA |  | FAM |
| *S. agalactiae* | 161 | AAGAAGTATTAGCACAAGAGCAAGC | CP019979.1 | N/A |
|  |  | GCTGGTGATACTGTTGTTRACT |  | FAM |
| MuV | 181 | GGTTGGATYGGACTTGTGTT | KF170916.1 | N/A |
|  |  | TGAGCAACGACTGGCAAA |  | FAM |
| *Nocardia* | 189 | GCCAACAGTGAAACGAGGT | KY417121.1 | N/A |
|  |  | CGGCRTYCTTCACCTTCA |  | FAM |
| HHV6 | 212 | CACCAGTATGTTCCACACCAAG | KY290185.1 | N/A |
|  |  | GTGCGTCTGGATGAAGAGGATT |  | FAM |
| Hu_DNA | 237 | GGTGTTTGCAGATTTGGACCT |  | N/A |
|  |  | CGCTAGGAATCAGACCAACACCTC |  | FAM |
| *A. baumanii* | 246 | GTTGTGAGTGATGCTGATGGG | CP020584.1 | N/A |
|  |  | TGACAAGACGACCAGACTGAT |  | FAM |
| *C. laurentii* | 253 | TGGAGGAGGCAGATCAAGG | KF036380.1 | N/A |
|  |  | GTGAAGATCGTCGGCAGGAA |  | FAM |
| *H. influenzae* | 269 | TCACCTGCATAACGCATAGGA | CP020411.1 | N/A |
|  |  | GTAACGGCATTGGTCAATACAGT |  | FAM |
| *C. albidus* | 303 | CGGTATCCACACGAAYCCAAG | KF036751.1 | N/A |
|  |  | GTCTCCTAGTAATTGCTCCCATCC |  | FAM |
| IC | 313 | GTGGCCGCTTTTCTGGATTCAT |  | N/A |
|  |  | TGAAGGCACAGTCGAGGCTG |  | FAM |

*H. influenzae: Haemophilus influenzae*, *L. monocytogenes: Listeria monocytogenes*, *S. agalactiae: Streptococcus agalactiae*, HHV-6: *human herpes virus type 6*, MuV: *mumps virus*, AB: *Acinetobacter baumannii*, *C. laurentii: Cryptococcus laurentii* and *C. albidus: Cryptococcus albidus*. Hu-RNA: Human RNA (B2M), Hu_DNA: Human DNA (RNaseP), IC: internal control.

Table S3. Thermal Cycling Conditions

| **Temperature** | **Reaction Time** | **No. of Cycles** |
| --- | --- | --- |
| 25°C | 5 min. | 1 |
| 50°C | 30 min. | 1 |
| 95°C | 15 min. | 1 |
| 94°C | 30 sec. | 65→60°C, 1°C reduction in annealing temperature, total of 6 cycles |
| 65→60°C | 30 sec. |  |
| 72°C | 60 sec. |  |
| 94°C | 30 sec. | 29 |
| 60°C | 30 sec. |  |
| 72°C | 60 sec. |  |
| 72°C | 15 min. | 1 |
| 4°C | Holding to CE | 1 |

Table S4. The Limit of Detection for ME Pathogens by AFA-Based Assay

| Target | Limit of Detection (copies/µL) | Limit of Detection (copies/rxn) |  |
| --- | --- | --- | --- |
| HCMV | 3 | 15 |  |
| EBV | 3 | 15 |  |
| HSV-1 | 13 | 65 |  |
| HSV-2 | 13 | 65 |  |
| VZV | 14 | 70 |  |
| EV | 16 | 80 |  |
| HHV6 | 17 | 85 |  |
| MuV | 18 | 90 |  |
| *M. tuberculosis* | 4 | 20 |  |
| *N. meningitidis* | 5 | 25 | |
| *S. pneumoniae* | 10 | 50 | |
| *E. coli* | 10 | 50 | |
| *Staphylococcus* | 16 | 80 | |
| *A. baumanii* | 16 | 80 | |
| *M. pneumoniae* | 16 | 80 |  |
| *H. influenzae* | 19 | 95 |  |
| *L. monocytogenes* | 20 | 100 |  |
| *S. agalactiae* | 22 | 110 |  |
| *Nocardia* | 24 | 120 |  |
| *C. neoformans* | 10 | 50 |  |
| *C. laurentii* | 12 | 60 |  |
| *C. albidus* | 15 | 75 |  |

Table S5. Baseline Characteristics of 170 ME

| Characteristic | Bacterial^§^ | Viral^§^ | Fungi^§^ | Tuberculous^§^ | Undefined^§^ | Total |
| --- | --- | --- | --- | --- | --- | --- |
|  | n=21 | n=61 | n=14 | n=43 | n=31 |  |
| Age (years) | 43 (17-68) | 43 (14-76) | 43 (16-78) | 31 (13-80) | 51 (5-71) | 42 (16-80) |
| Sex, n (%) |  |  |  |  |  |  |
| Male, n (%) | 12 (57.1) | 35 (57.4) | 7 (50.0) | 25 (58.1) | 22 (71.0) | 101 (59.4) |
| Female | 9 (42.9) | 26 (42.6) | 7 (50.0) | 18 (41.9) | 9 (29.0) | 69 (40.6) |
| Race |  |  |  |  |  |  |
| Han nationality | 19 (90.4) | 60 (98.4) | 14 (100) | 30 (69.8) | 28 (90.3) | 151 (88.8) |
| Tibetan | 1 (4.8) | 1 (1.6) | 0 | 12 (27.9) | 2 (6.5) | 16 (9.4) |
| Other | 1 (4.8) | 0 | 0 | 1 (2.3) | 1 (3.2) | 3 (1.8) |
| Hypertension, n (%) | 2 (9.5) | 9 (14.8) | 4 (28.6) | 4 (9.3) | 2 (6.5) | 21 (12.4) |
| Diabetes, n (%) | 1 (4.8) | 1 (1.6) | 1 (7.1) | 3 (7.0) | 0 | 6 (3.5) |
| Immunosuppressed^‡^, n (%) | 3 (14.3) | 2 (3.3) | 4 (28.6) | 3 (6.52) | 2 (6.5) | 14 (9.3) |
| HIV/AIDS, n (%) | 1 (4.8) | 1 (1.6) | 3 (21.4) | 1 (2.3) | 0 | 6 (3.5) |
| Lung infection, n (%) | 11 (52.4) | 29 (47.5) | 10 (71.4) | 17 (39.5) | 13 (41.9) | 80 (47.1) |
| Hypokalemia, n (%) | 5 (23.8) | 14 (23.0) | 12 (85.7) | 19 (44.2) | 12 (38.7) | 62 (36.5) |
| Hypoproteinemia, n (%) | 2 (9.5) | 5 (8.2) | 2 (14.3) | 9 (20.9) | 9 (29.0) | 27 (15.9) |
| Pulmonary tuberculosis | 2 (9.5) | 1 (1.6) | 0 | 17 (39.5) | 2 (6.5) | 20 (11.8) |
| Clinical characteristics |  |  |  |  |  |  |
| Fever, n (%) | 15 (71.4) | 35 (57.4) | 4 (28.6) | 33 (76.7) | 15 (48.4) | 102 (60) |
| Temperature >38.5°C, axillary | 10 | 21 | 3 | 23 | 8 | 75 |
| Vomiting, n (%) | 4 (19.0) | 6 (9.8) | 3 (21.4) | 8 (18.6) | 5 (16.1) | 26 (15.3) |
| Headache, n (%) | 10 (47.6) | 35 (57.4) | 11 (78.6) | 34 (79.1) | 16 (51.6) | 106 (62.4) |
| Nuchal rigidity or stiff neck | 9 (42.9) | 19 (31.1) | 5 (35.7) | 26 (60.5) | 9 (29.0) | 68 (40%) |
| Meningeal irritation, n (%) | 3 (14.3) | 8 (13.1) | 1 (7.1) | 9 (20.9) | 5 (16.1) | 26 (15.3) |
| Mental disorders, n (%) | 1 (4.8) | 12 (19.7) | 0 | 2 (4.7) | 1 (3.2) | 16 (9.4) |
| Seizures, n (%) | 2 (9.5) | 9 (14.8) | 29 (14.3) | 4 (9.3) | 2 (6.5) | 46 (27.1) |
| Confusion, n (%) | 3 (14.3) | 16 (26.2) | 2 (14.3) | 12 (27.9) | 8 (25.8) | 41 (24.1) |
| Vision loss, n (%) | 3 (14.3) | 0 | 2 (14.3) | 1 (2.3) | 0 | 6 (3.5) |
| Sepsis, bacteremia, n (%) | 4 (19.0) | 2 (3.3) | 2 (14.3) | 0 | 0 | 8 (4.7) |
| Sinusitis or otitis, n (%) | 9 (42.9) | 26 (42.6) | 4 (28.6) | 15 (34.9) | 19 (61.3) | 73 (43.0) |
| Time interval from illness onset to hospitalization (days) | 24 (4-49) | 16 (4-67) | 29 (14-58) | 25 (7-102) | 26 (2-66) | 22 (2-102) |
| Prognosis, n (%) |  |  |  |  |  |  |
| Death | 2 | 0 | 2 | 0 | 0 | 4 (2.4) |
| Disability | 8 | 10 | 5 | 11 | 8 | 42 (24.7) |
| Good recovery | 11 | 51 | 7 | 32 | 23 | 124 (72.9) |

*Data are presented as number (percentage) or median (range).

‡Immunosuppressed: Patients with long-term use of hormone drugs, AIDS and other immune suppressors.

§The precision of the sub-group definition was as follows:

**AS for bacterial ME:** a person of any age who experienced acute onset of fever with changes in mental status and/or meningeal signs (e.g., neck stiffness or headache) if the laboratory test results demonstrated at least 1 of the following: a turbid appearance of CSF; leukocytosis (>100 cells/mm^3^; reference 0–8 cells/mm^3^); or leukocytosis (10–100 cells/mm^3^) and an elevated protein level (>100 mg/dL; reference 20-40 mg/dL) or decreased glucose (<40 mg/dL; reference 50–80 mg/dL). These signs are consistent with the World Health Organization (WHO) PBM case definition.

**AS for viral ME:** Major criterion (required): patient presenting to medical attention with an altered mental status (defined as decreased or altered level of consciousness, lethargy, or personality change) lasting ≥24 h with no alternative cause identified.

Minor Criteria (2 required for possible encephalitis; ≥3 required for probable or confirmed encephalitis): documented fever ≥38° C (100.4°F) within the 72 h before or after presentation; generalized or partial seizures not fully attributable to a preexisting seizure disorder; new onset of focal neurologic findings; CSF WBC count ≥5/cubic mm; abnormality of the brain parenchyma on neuroimaging suggestive of encephalitis that is either new from prior studies or appears acute in onset; and abnormality on electroencephalography that is consistent with encephalitis and not attributable to another cause.

**AS for TBM**: A diagnosis of definite tuberculous meningitis should be made when one or more of the following criteria are met: acid-fast bacilli (AFB) seen in the CSF, *M. tuberculosis* cultured from the CSF, or a CSF *M. tuberculosis*-positive commercial nucleic acid amplification test from a patient who presents with symptoms or signs suggestive of meningitis; AFB seen in the context of histological changes consistent with tuberculosis in the brain or spinal cord together with suggestive symptoms or signs and CSF changes; or visible meningitis (on autopsy).

Probable tuberculous meningitis: clinical meningitis and one or more of

the following:

i) Suspected active pulmonary tuberculosis based on CXR

ii) AFB found in any sample other than the CSF

iii) Clinical evidence of other extrapulmonary tuberculosis

Possible tuberculous meningitis: clinical meningitis and four or more of

the following:

i) History of tuberculosis

ii) Predominance of lymphocytes in the CSF

iii) Illness of more than 5 days in duration

iv) CSF to blood glucose ratio less than 0.5

v) Altered consciousness

vi) Yellow CSF

vii) Focal neurological signs

**AS for CM:** Symptoms and signs of meningitis including one or more of the following: headache, vomiting, fever, neck stiffness, convulsions, focal neurological deficits, and altered mentation. Laboratory assessments for CM include CSF culture, CSF India ink testing, and CSF cryptococcal antigen testing (CrAg).

Table S6. Laboratory Results and Examination of ME Patients

| Laboratory results and examination | Bacterial (n=21) | Viral (n=61) | Fungi (n=14) | Tuberculous (n=43) | Undefined  (n=31) | Total |
| --- | --- | --- | --- | --- | --- | --- |
|  | Group 1 | Group 2 | Group 3 | Group 4 | Group 5 |  |
| Opening pressure (mmH_2_O) | 200 (30-280) | 120 (61-350) | 240 (190-320) | 210 (90-350) | 180 (75-350) | 180 (61-380) |
| Opening pressure>250 mmH_2_O | 4 (19.0) | 7 (11.5) | 6 (42.9) | 11 (25.6) | 10 (32.3) | 32 (18.8) |
| Closing pressure (mmH_2_O) | 115 (10-200) | 65 (14-195) | 155 (50-230) | 100 (30-260) | 100 (35-200) | 90 (14-260) |
| CSF clear appearance | 12/19 (63.2) | 46/54 (85.2) | 10/13 (76.9) | 36/42 (85.7) | 20/30 (66.7) | 124/158 (78.5) |
| CSF Leucocyte Count (*10^6^/L) | 670 (0-20000) | 20 (0-420) | 34 (0-130) | 95 (0-2200) | 95 (0-2480) | 70 (0-20000) |
| Monocytes | 20 (0-100) | 95 (0-100) | 80 (0-100) | 85 (0-100) | 78 (0-100) | 85 (0-100) |
| Leukocytes | 85 (0-100) | 10 (0-85) | 20 (0-65) | 17 (0-98) | 22 (2-100) | 20 (0-100) |
| CSF Glucose (mmol/L) | 2.01 (0.02-7.96) | 3.24 (0.33-5.06) | 2.65 (2.03-5.00) | 1.92 (0.58-7.87) | 2.70 (0.50-5.06) | 2.74 (0.02-7.96) |
| <2.5 mmol/L | 13/19 (68.4) | 6/ 54 (11.1) | 4/13 (30.8) | 16/4 2 (38.1) | 16/30 (53.3) | 55/158 (34.8) |
| CSF Protein | 1.15 (0.49-21.00) | 0.61 (0.16-32.00) | 0.59 (0.22-0.96) | 1.45 (0.13-8.30) | 0.99 (0.24-31.00) | 0.81 (0.13-32.00) |
| >0.45 g/L | 19/19 (100.0) | 37/54 (68.5) | 9/1 3 (69.2) | 41/ 4 2 (97.6) | 27/30 (90.0) | 136/158 (86.1) |
| CSF chlorinate | 121 (106.7-138.6) | 124 (77.60-144.50) | 123.50 (114.6-137.9) | 115.30 (11.40-134.10) | 118.35 (11.40-141.30) | 121.9 (11.40-144.50) |
| <120 mmol/L | 10/19 (52.6) | 14/54 (18.5) | 2/13 (15.4) | 26/42 (61.9) | 15/30 (50.0) | 67/158 (42.4) |
| CSF IgG synthesis rate | 51.25 (14.74-228.23) | 3.104 (0-290) | 7.289 (6.42-59.56) | 21.36 (0.00-142.47) | 7.354 (0.00-72.03) | 12.430 (0.00-290.0) |
| ESR | 37 (2-79) | 23 (2-80) | 55 (15-63) | 26 (2.0-91) | 21 (6.0-66.0) | 25 (2.0-91) |
| Glucose level (mmol/L) | 5.07 (3.85-14.06) | 5.29 (3.89-9.99) | 7.22 (4.19-9.60) | 5.77 (4.08-14.59) | 5.86 (4.06-13.37) | 5.39 (3.85-14.59) |
| CSF G/Serum G | 0.44 (0.01-0.80) | 0.59 (0.06-0.91) | 0.58 (0.25-0.62) | 0.35 (0.10-0.72) | 0.42 (0.12-0.48) | 0.48 (0.01-0.91) |
| Serum leukocyte count (cells/µL) | 5.69 (1.91-17.40) | 6.84 (3.52-18.64) | 9.66 (4.80-18.96) | 6.75 (2.31-19.57) | 8.59 (4.53-19.57) | 7.26 (2.31-20.14) |
| CRP, C-reactive protein (mg/L) | 27.35 (2.52-122) | 13.80 (1.31-233) | 9.10 (2.94-208) | 8.21 (1.00-197.00) | 8.34 (1.31-122.0) | 9.10 (1.00-233) |
| PCT, Procalcitonin (ng/mL) | 0.18 (0.02-56.51) | 0.20 (0.02-2.50) | 0.10 (0.03-1.50) | 0.09 (0.02-4.10) | 0.15 (0.02-1.60) | 0.15 (0.02-56.51) |
| Head CT taken | 11 | 29 | 5 | 21 | 13 | 75 (47.47) |
| Abnormal | 4 (36.4) | 10 (34.5) | 1 (20) | 9 (42.9) | 8 (61.5) | 23/75 (30.7) |
| Brain MRI taken | 16 | 45 | 8 | 36 | 19 | 109 (68.13) |
| Abnormal | 11 (68.8) | 15 (33.3) | 6 (75) | 30 (76.9) | 17 (89.50 | 58 (53.2) |
| Meningeal enhancement | 5 (31.3) | 9 (20.0) | 5 (62.5) | 26 (72.2) | 9 (47.4) | 45 (41.28) |

Table S7. Detailed information for samples with inconsistent results

|  |  | Demographic data | CSF parameter |  |  |  | |  |  | |  |  |
| --- | --- | --- | --- | --- | --- | --- | --- | --- | --- | --- | --- | --- |
| Patient. no | Target | Age, sex | CSF Appear-ance | WBC | Cell types * | Glucose | | Protein | Clinical features and signs | | Final diagnosis |  |
| 43 | *E. coli* | 49, M | Clear | 90 | 54, L | 1.48 | | 1.66 | Fever, Nuchal rigidity | | TBM, Confusion, Cerebral infarction |  |
| 77 | *E. coli* | 39, F | Clear | 0 |  | 3.30 | | 0.43 | Headache | | CNS, G- bacilli |  |
| 139 | *E. coli* | 19, F | Clear | 0 |  | 1.88 | | 0.61 | Fever | | BM |  |
| 63 | *Staphylococcus* | 23, M | Clear | 0 |  | 3.2 | | 0.33 | Fever | | Viral ME |  |
| 65 | *Staphylococcus* | 43, M | Clear | 100 | 80, N | 3.57 | | 0.52 | Headache, diplopia | | BM, anal fistula, postoperative abducens nerve paralysis |  |
| 108 | *A. baumanii* | 29, M | Clear | 2 | ND | 5.31 | | 0.27 | Seizures, dysphasia, mental disorders | | Viral ME |  |
| 1 | *S. pneumonia* | 20, M | Cloudy | 150 | 80, L | 1.55 | | 0.84 | Fever, vomiting, headache, sinusitis or otitis, paranasal sinusitis | | CNS, acute suppurative tonsillitis |  |
| 27 | *S. pneumoniae* | 58, M | Clear | 2 | ND | 3.54 | | 0.24 | Fever, headache, otitis, dysphasia, mental disorders | | CNS |  |
| 153 | *N. meningitidis* | 16, M | Clear | 200 | 79, N | 1.97 | | 1.47 | Fever, headache, chills, nuchal rigidity, meningeal irritation | | BM |  |
| 5 | *M. tuberculosis* | 68, M | Xanthochromic | 2200 | 97, N | 3.41 | | 0.70 | Headache, tinnitus | | TBM, genital tuberculosis, IGRA (+) |  |
| 159 | *M. tuberculosis* | 15, M | Clear | 0 |  | 3.52 | | 0.94 | Headache | | TBM, hyperuricemia, abnormal liver function, IGRA (+) |  |
| 87 | EBV | 34, F | Clear | 160 | 90, N | 1.60 | | 2.06 | Fever, nuchal rigidity, meningeal irritation | | TBM, Cushing's syndrome, hyperuricemia, lung infection, tuberculosis |  |
| 156 | EBV | 16, M | Clear | 150 | 98, N | 2.37 | | 1.01 | Headache, fever, nuchal rigidity | | TBM |  |
| 12 | EV | 61, M | Clear | 170 | 75, N | 1.79 | | 1.50 | Fever, headache, vomiting, nuchal rigidity, meningeal irritation | | TBM, diabetes, Tuberculosis, hypoproteinemia, chronic glomerulonephritis |  |
| 8 | *Cryptococcus* | 26, M | Clear | 40 | 97, L | 1.97 | | 0.82 | Headache, nuchal rigidity, dermatitis | | CM, AIDS, lung infection |  |
| 21 | *C. neoformans* | 43, M | Clear | 120 | 100, L | 2.34 | | 0.67 | Fever, headache, vomiting | | CM, sepsis, lung infection, upper gastrointestinal hemorrhage, hemorrhagic shock, hypoproteinemia, hypokalemia |  |
| 55 | *C. neoformans* | 17, M | Xanthochromic | 130 | 65, L | 2.03 | | 0.62 | Fever, vision loss, nuchal rigidity | | CM, eosinophilia, |  |
| 122 | *C. neoformans* | 21, M | Clear | 0 |  | 3.77 | | 0.22 | Fever, headache | | CM, AIDS, upper respiratory tract infection, seizure, paranasal sinusitis |  |
| 129 | *M. Tuberculosis, N. meningitidis* and EBV | 18,M | Clear | 200 | 21, N | 1.97 | 1.47 | Fever, headache, chills,  nuchal rigidity, meningeal irritation, disturbance of consciousness, hydrocephalus | | TBM, TB PCR (+) tuberculosis, IGRA (+), serum EBV PCR (+). | |  |

*** Values are % neutrophils (N) or % lymphocytes (L) unless otherwise indicated. ND, not determined.

Table S8. Detailed information for samples with consistent results

|  |  | Demographic data | CSF parameters | | | | |  |  |
| --- | --- | --- | --- | --- | --- | --- | --- | --- | --- |
| Patient. no | Target | Age, sex | CSF Appear-ance | WBC | Cell types* | Glucose | Protein | Clinical features and signs  Final diagnosis  Final resolution of AFA result | Laboratory findings |
| 7 | *E. coli* | 61, M | Xanthochromic | 800 | 98, N | 2.30 | 0.71 | CNS, subarachnoid hemorrhage, presenting with fever, vomiting, nuchal rigidity, meningeal irritation, paranasal sinusitis |  |
| 36 | *Staphylococcus* | 52, F | Bloody | 630 | 80, N | 0.88 | 2.83 | BM, SLE, anemia, presenting with fever, meningeal irritation, nuchal rigidity, confusion |  |
| 68 | *A. baumanii* | 42, M | Xanthochromic | 20000 | 85, N | 0.11 | 8.50 | BM, death, presenting with fever, nuchal rigidity, septic shock, hydrocephalus, cerebral hematoma |  |
| 89 | *A. Baumanii* and CMV | 31, M | Bloody | 700 | 88, N | 0.03 | 21.00 | CNS (*A. baumanii),* intracranial surgery, cerebral hemorrhage, hydrocephalus, electrolyte disorder, headache | *A. Baumanii* CSF culture (+),CMV CSF IgM antibodies demonstrating at least a 4-fold increasing |
| 134 | *A. baumanii* | 20, M | Clear | 30 | 98, N | 2.76 | 6.70 | CNS (*A. baumanii),* cerebral hemorrhage, hydrocephalus, Urinary tract infection, presenting with fever, nuchal rigidity, confusion |  |
| 146 | *A. baumanii* | 16, M | Xanthochromic | 2480 | 95, N | 2.11 | 4.80 | CNS (*A. baumanii)* |  |
| 42 | *M. tuberculosis* | 50, M | Clear | 0 | ND | 4.03 | 32.00 | TBM, cerebral infarction, hypertension | PCR (+) |
| 56 | *M. tuberculosis* | 49, F | Clear | 100 | 84, L | 1.99 | 4.00 | TBM, urinary tract infection, abnormal liver function, hypoproteinemia, presenting with fever, headache, meningeal irritation | PCR (+) |
| 59 | *M. tuberculosis* | 47, M |  |  |  |  |  | TBM, AIDS, lung infection, presenting with fever, headache and mental disorders | PCR (+), IGRA (+) |
| 70 | *M. tuberculosis* | 42, F | Clear | 20 | ND | 1.14 | 1.79 | TBM, tuberculosis, electrolyte disturbance, presenting with fever, cough, nuchal rigidity, | PCR (+), CSF culture (+), IGRA (+) |
| 72 | *M. tuberculosis* | 39, F | Clear | 260 | 95, L | 1.05 | 1.31 | TBM, presenting with nuchal rigidity, meningeal irritation | PCR (+), IGRA (+) |
| 86 | *M. tuberculosis* | 37, M | Xanthochromic | 2200 | 97, N | 3.41 | 0.70 | TBM, genital tuberculosis, presenting with headache, tinnitus | PCR (+), IGRA (+) |
| 88 | *M. tuberculosis* | 31, M | Clear | 40 | 83, L | 1.72 | 8.30 | TBM, tuberculosis, tuberculous peritonitis, presenting with headache, fever, vomiting, nuchal rigidity | PCR (+), IGRA (+) |
| 110 | *M. tuberculosis* | 29, M | Clear | 140 | 94, L | 0.93 | 4.28 | TBM, lung infection, polyserositis, hepatic cyst, presenting with headache, nuchal rigidity | PCR (+), IGRA (+) |
| 144 | *M. tuberculosis* | 18, M | Clear | 0 |  | 3.57 | 0.13 | TBM, tuberculosis, tuberculous peritonitis, hypoproteinemia, electrolyte disturbance, tinnitus presenting with headache, fever, chills, nuchal rigidity | PCR (+), CSF culture (+), IGRA (+) |
| 160 | *M. tuberculosis* | 15, F | Clear | 40 | 90, L | 2.24 | 2.23 | TBM, tuberculosis. presenting with headache, fever, nuchal rigidity | PCR (+), CSF culture (+), IGRA (+) |
| 164 | *M. tuberculosis* | 14, F | Clear | 50 | 85, L | 0.72 | 1.25 | TBM, tuberculosis, cerebral infarction, peritoneal tuberculosis, presenting with headache, fever, nuchal rigidity | PCR (+), IGRA (+), pathological biopsy: acid-fast bacillus |
| 128 | CMV | 20, M | Clear | 90 | 95, L | 1.47 | 3.01 | Viral ME, lung infection, stomatitis, AIDS, paranasal sinusitis, presenting with headache, fever | Sequencing (+) |
| 165 | CMV | 7, M | Bloody | 0 |  | 3.44 | 0.76 | ME (undefined), hydrocephalus, lung infection presenting with headache and confusion | Sequencing (+) |
| 57 | EBV | 47, M | Clear | 0 |  | 2.65 | 0.41 | CNS (EB), Serous cavity effusion, gout, splenomegaly presenting with headache, fever, chills | Sequencing (+), EB PCR (+) |
| 81 | EBV | 39, F | Bloody | 420 | 86, L | 3.76 | 5.62 | CNS, subarachnoid hemorrhage, hyperlipidemia, lung infection presenting with headache, fever, slow in reacting, nuchal rigidity, meningeal irritation | Sequencing (+) |
| 169 | EBV | 46, F | Clear | 0 |  | 3.39 | 0.51 | Viral ME, hypertension, otitis, presenting with headache, fever, nuchal rigidity, meningeal irritation | Sequencing (+) |
| 158 | VZV | 15, F | Clear | 0 |  | 4.84 | 0.61 | Viral ME, ethmoid sinus and maxillary sinusitis, presenting fever and vomiting | Sequencing (+) |
| 121 | MuV | 24, F | Xanthochromic | 30 | 69, L | 2.52 | 1.07 | Viral ME, hydrocephalus, hypokalemia, hypoproteinemia | Sequencing (+) |
| 116 | HSV-1 | 27, M | Clear | 156 | 72, L | 2.29 | 1.18 | Viral ME, hypokalemia, leucopenia, anemia, presenting fever and vomiting, nuchal rigidity, meningeal irritation | Sequencing (+) |
| 117 | HSV-1 | 26, M | Clear | 0 |  | 3.61 | 0.67 | Viral ME, lung infection, hypertension, anemia, cerebral hemorrhage, hypoproteinemia, presenting with fever and confusion | Sequencing (+) |
| 6 | EV | 64, F | Clear | 0 |  | 3.39 | 0.25 | Viral ME, mental disorders | Sequencing (+) |
| 10 | EV | 62, M |  |  |  |  |  | Viral ME, language barrier | Sequencing (+) |
| 9 | *C. neoformans* | 62, F | Clear | 80 | 95, L | 2.83 | 0.52 | CM, hydronephrosis, lung infection, hypokalemia, presenting with headache, fever, meningeal irritation and seizures | CSF culture (+), Jincheng ink (+) |
| 16 | *C. neoformans* | 61, F | Clear | 10 | ND | 2.19 | 0.64 | CM, lung infection, hypokalemia, hypothyroidism, anemia, mastoiditis, presenting with headache and nuchal rigidity | CSF culture (+), Jincheng ink (+) |
| 33 | *C. neoformans* | 53, M | Clear | 18 | ND | 3.84 | 0.35 | CM, cerebral hernia, hepatitis B, presenting with headache, nuchal rigidity and meningeal irritation | CSF culture (+), Jincheng ink (+) |
| 35 | *C. neoformans* | 53, M | Bloody | 50 | 100, L | 2.24 | 0.76 | CM, hypoproteinemia, presenting with headache | CSF Jincheng ink (+), CSF culture (+) in a former CSF sample |
| 38 | *C. neoformans* | 50, F | Clear | 2 | ND | 2.41 | 0.59 | CM, hyperlipemia, abdomen tuberculosis, presenting with headache | CSF culture (+), Jincheng ink (+) |
| 102 | *C. neoformans* | 29, M | Clear | 240 | 78, L | 2.66 | 1.75 | CM, hypertension, presenting with headache and fever | CSF culture (+) |
| 127 | *C. neoformans* | 22, M | Clear | 40 | 80, L | 4.15 | 0.62 | CM, AIDS, pneumocystis pneumonia (PCP), hypokalemia, presenting with headache and fever | CSF culture (+) |

*** Values are % neutrophils (N) or % lymphocytes (L) unless otherwise indicated. ND, not determined.
